# High-concentrate diet supplemented with hydrolysable tannin improves the slaughter performance, intestinal antioxidant ability and barrier function of fattening lambs

**DOI:** 10.3389/fvets.2024.1464314

**Published:** 2024-10-28

**Authors:** Jian Ma, Tao Li, Lu Lin, Yuezhang Lu, Xi Chen, Sibing Li, Chen Wei, Chunmei Du, Fuquan Yin, Guang Cao, Shangquan Gan

**Affiliations:** ^1^College of Coastal Agricultural Sciences, Guangdong Ocean University, Zhanjiang, China; ^2^Tie Qi Li Shi Feed Co., Ltd., Chengdu, China

**Keywords:** hydrolysable tannin, lamb, slaughter performance, intestinal barrier junction, antioxidant ability

## Abstract

The objective of current experiment was to study the potential influence of hydrolysable tannin supplementation on slaughter performance, meat quality, intestinal digestive enzyme activity, antioxidant ability and barrier function in fattening lambs. In total, 36 male Hu sheep lambs with similar body weight (15.83 ± 0.48 kg) and days in age (55 ± 2 d) were randomly assigned to one of three groups of 12 animals each: control without tannin (CON) and tannin supplementation groups (TA1, 3 g/d per lamb; TA2, 6 g/d per lamb). All the lambs were reared in individual hutches, and the experiment lasted for 60 d. On d 61, 8 lambs from each group were randomly selected to slaughter. Results showed that the serum diamine oxidase and lipopolysaccharide contents of TA2 group were higher (*p* < 0.05) than those of CON group. Compared with CON group, the carcass weight and intramuscular fat content of lambs in TA1 group were increased (*p* < 0.05) and the meat shear force was decreased (*p* < 0.05). The trypsin activity in the jejunum and ileum of TA1 group was higher (*p* < 0.05) than that of CON and TA2 groups. Also, tannin supplementation significantly increased (*p* < 0.05) the level of jejunal and ileal total antioxidant capacity and reduced (*p* < 0.05) the jejunal malondialdehyde concentration in lambs. The jejunum and ileum of TA1 lambs showed reduced (*p* < 0.05) tumor necrosis factor-alpha and increased (*p* < 0.05) interleukin-10 mRNA levels compared with CON lambs. In the jejunum, the secretory immunoglobulin A content of TA1 group was higher (*p* < 0.05) than that of CON and TA2 groups. Lambs supplemented with tannin at the level of 3 g/d increased (*p* < 0.05) the gene expressions of *claudin-1*, *claudin-4* and zonula occludens-1 in the jejunum when compared to those of CON and TA2 groups. In summary, tannin supplementation at the level of 3 g/d per animal can improve the production performance and intestinal function of fattening lambs fed a high-concentrate diet.

## 1 Introduction

With the improvement of living standard, the demand of meat consumption, especially mutton, has gradually increased. In some countries, such as China and Mongolia, mutton is an important food source for humans because it can supply high quality nutrients, which mainly include protein, fatty acid, mineral substance and vitamin (1). Recently, the increased consumption of mutton has significantly stimulated the development of sheep breeding industry. In some regions of the world, the land resources are limited. Moreover, the requirement for ecological conservation and restoration of grassland is urgent. Thus, in sheep production, the traditional grazing pattern has gradually changed into large-scale intensive farming. Nevertheless, under the feeding pattern of intensive feeding, the living space is limited and feed type is changed, which can reduce the antioxidant ability and meat quality as well as increase the mortality and morbidity of sheep. These negative effects mentioned earlier severely restrict the sustainable development of mutton sheep industry (2, 3). In the sheep production, because of high meat quality and fast growth rate of lambs, the lamb fattening is a common production pattern, which can shorten the time to market and increase economic benefits. However, the function of immune system and gastrointestinal tracts is relatively lower during the lamb stage. Moreover, in order to meet the requirements of energy and other nutrients, the fattening lambs are often provided high-concentrate diet for maintaining healthy growth and productivity. Long-term feeding of high-concentrate further aggravates the damage on the gastrointestinal tracts and oxidative stress of lambs, which finally can induce systemic inflammation and some disorders in lambs (4). Therefore, relieving oxidative stress and improving intestinal function of fattening lambs are of great significance in intensive breeding of mutton sheep industry.

In recent years, the application of plant-derived natural active material in animals production has received extensive attention. Bioactive compounds in natural active material including polyphenols have shown to enhance intestinal barrier function and antioxidant ability as well as improving nutrient absorption and digestion, which have positive influence on growth and production performance of ruminants (5–7). As a plant polyphenol, the utilization of tannin has become more and more extensive due to its multiple biological activities, mainly including antioxidative, anti-inflammatory and antibacterial functions (8, 9). With the prohibition of use of antibiotics in the diet, the tannin, a green feed additive, has been utilized in ruminants production as an alternative to in-feed antibiotics. A previous study in Holstein Calves found that dietary supplementation of tannin significantly increased the dry matter intake and average daily gain (10). In transition dairy cows, supplementation of 1.97% chestnut tannin in the diet can reduce the damage degree of liver cell membrane, protect thyroid gland and improve the synthesis of triiodothyronine and thyroxine, which were helpful for preventing various postpartum metabolic diseases caused by excessive body fatty mobilization (11). According to the chemical structure, tannins can be divided into hydrolysable tannin and condensed tannin. Among them, hydrolysable tannin is deemed to be one of effective antioxidants, which can prevent cell damage by scavenging free radical (12). Due to high level of polyphenols, the tannin can be used to alleviate various physiological stress. In heat-stressed lambs, dietary supplementation of tannin increased the yellowness and lightness values of meat and the concentrations of superoxide dismutase (SOD) and glutathione peroxidase (GSH-Px) in serum and liver, while decreased the malondialdehyde (MDA) content in muscle, serum and liver, and finally improved the meat quality and antioxidant capacity of heat-stressed lambs (13). However, the potential positive influences of tannin on meat quality and antioxidant ability of fattening lambs fed a high-concentrate diet still need to be fully investigated.

As vital organ for diet digestion and nutrients absorption, the integrity of gastrointestinal tracts is tightly linked with immune function and production performance of animals (14). The long-term feeding high-concentrate ration leads to gastrointestinal tracts acidosis and several metabolic disorders, which can damage the barrier function and induce inflammatory response of gut (15). Tannin can condense with proteins through hydrophobic or hydrogen bonds to form stable tannin-protein complexes, which is the main reason for the intestinal astringent effect of tannins. Thus, tannin plays an important role in improving intestinal health of animals (16). Unfortunately, the information of effects of tannin as a feed additive on intestinal barrier function and inflammatory response of fattening lambs that are fed high-concentrates remains scarce. In addition, because the rumen and large intestine are the main organ for fermentation of high levels of grain, most of researches in ruminants fed high-concentrates focus on the rumen, cecum and colon (17, 18). The small intestine is mainly responsible for nutrients digestion and absorption. Based on previous studies, we hypothesized that tannin supplementation may improve the small intestinal health of fattening lambs. Therefore, the current research was carried out to explore the effects of tannin on slaughter performance, meat quality, intestinal digestive enzyme activity, antioxidant ability and barrier function of fattening lambs fed a high-concentrate diet.

## 2 Materials and methods

### 2.1 Experimental design and lambs management

In this experiment, a total of 36 male Hu lambs (body weight = 15.83 ± 0.48 kg; days in age = 55 ± 2 d; mean ± standard deviation) were selected. After labeling with ear tags, the selected animals were randomly assigned to one of three group with 12 lambs in each group. The lambs in control group were fed basal ration with no tannin supplementation (CON), and the lambs in experimental treatments were fed basal ration plus 3 g/d (TA1) and 6 g/d (TA2) per lamb tannin (hydrolysable tannin ≥78%; Wufeng Chicheng Biotechnology Co., Ltd., Yichang, China) respectively. The supplemented dosage of tannin were based on the previous research in heat-stressed lambs (13) and recommendation of manufacturer for ruminants.

All experimental animals were allocated to individual hutches with one lamb in each hutch (2 m × 1 m). The lambs were provided a same experimental ration twice daily (08:30 and 18:30) at 105% of free access to intake, and had access to clean water that was freely consumed during the experimental period. At morning feeding, the tannin was weighted daily, and then hand-mixed into basal diet to feed animals and ensure complete intake. The basal diet of fattening lambs with a 70:30 concentrate to roughage was designed according to the NRC (19). The alfalfa hay and corn straw were used as roughage in the diet, and the concentrates were mainly composed of corn, soybean meal, wheat bran and cottonseed meal. Supplementary Table S1 shows feed compositions and nutrients levels of experimental diet. The nutritional levels of diet were analyzed according to the AOAC procedures (20). The experiment was carried out with a 10 d of adaption stage followed by 60 d of formal feeding trial.

### 2.2 Blood samples collection and analysis and determination of slaughter performance

Before morning feeding on d 60 of the feeding trial, the blood samples of each animal were taken from the jugular vein using 5 mL vacuum tubes (Shengcixing Trading Co., Ltd., Hengshui, China). Next, serum samples were isolated by centrifuging blood at 3200 rpm for 12 min and collected in 1.5 mL centrifugal tubes (−20°C) for subsequent analysis. After thawing, the contents of permeability indexes including diamine oxidase (DAO), D-lactic acid (D-LA) and lipopolysaccharide (LPS) in serum samples were determined by commercial kits (Nanjing Jiancheng Bioengineering Institute, Nanjing, China) following the instructions.

On d 61, 8 lambs from each group were randomly selected to be fasted for 12 h, and the liveweight was recorded. Subsequently, lambs were slaughtered at a commercial slaughterhouse, and performed by professionals following the National Standard Operating Procedures (GB/T 43562–2023, sheep slaughtering, China). All lambs were given to three groups to finish the process of slaughter and samples collection within 1 day. After slaughter, the weight of hot carcass was obtained, and then the meat from the carcass was removed and weighed. The dressing percentage was calculated by dividing carcass weight by liveweight. Besides, the meat percentage was calculated by dividing meat weight by liveweight.

### 2.3 Samples collection of meat, intestinal digesta and tissue

After slaughter, a piece of *longissimus dorsi* muscle from the left side of carcass between the eighth and ninth rib were immediately collected. The collected meat was placed into sterile vacuum package (Hongbang Packing Products Co., Ltd., Huizhou, China), and then stored at an average temperature of 4°C for determination of meat quality. After opening the abdominal cavity of each lambs, the duodenum, jejunum and ileum were isolated with suture line to avoid digesta backflow. The digesta samples in the duodenum, jejunum and ileum were collected, put in 5 mL centrifugal tube and stored at −80°C for analysis of digestive enzyme activity. Subsequently, the digesta was removed from the small intestine, and the small intestine was washed with ice cold sterile phosphate buffer solution. The mucosal samples were obtained by scratching using a sterile glass microscope slide from the mid-portion of each segment of small intestine. After collection, the mucosal samples were put in sterile 5 mL centrifugal tube and stored at −80°C for following analysis.

### 2.4 Analysis of meat quality, digestive enzyme activity and intestinal antioxidant capacity

The pH value of *longissimus dorsi* muscle was measured at 45 min and 24 h post-mortem of each carcass by a pH meter installed with penetrating electrode (Honghui Instrument Equipment Co., Ltd., Shanghai, China). Meat color parameters including lightness (L*), redness (a*) and yellowness (b*) was measured with a meat colorimeter (NS800, Sanenshi Technology Co., Ltd., Shenzhen, China). The content of intramuscular fat (IMF) was analyzed reference to the procedures of previous study (21). Briefly, the lyophilized *longissimus dorsi* muscle samples were performed to ether extraction using diethyl ether at a Soxhlet extractor (Qiwei Instrument Co., Ltd., Hangzhou, China). After extraction, meat samples were dried with a forced-air oven at 55°C and reweighed to measure fatty loss. In addition, the analysis of drip loss, cooking loss and shear force was followed the procedures described by Hanoglu et al. (22). The result of meat drip loss (%) was obtained as the weight loss relative to initial weight, and the meat cooking loss (%) was calculated as the percentage of weight loss before and after cooking. The shear force value of *longissimus dorsi* muscle from each lambs was measured using a shear force instrument (Shengtai Instrument Co., Ltd., Jinan, China).

The digesta samples in the small intestine was used to analyze the activity of digestive enzymes including *α*-amylase, trypsin, chymotrypsin and lipase using the assay kits according to the operating instructions (Nanjing Jiancheng Bioengineering Institute, Nanjing, China). The 500 mg intestinal mucosa samples were homogenized on ice in 4.5 mL of normal saline. Subsequently, the mixture was used to centrifuge at 3500 rpm (4°C) for 15 min to collect supernatant. The obtained supernatant was analyzed for MDA and antioxidant enzyme concentrations, including GSH-Px, SOD and total antioxidant capacity (T-AOC) by using commercial kits (Nanjing Jiancheng Bioengineering Institute, Nanjing, China). Moreover, the concentration of secretory immunoglobulin A (SIgA) in the small intestinal mucosa was measured with a commercial kit (Nanjing Jiancheng Bioengineering Institute, Nanjing, China) following the specifications.

### 2.5 Quantitative real-time PCR for inflammatory factors and tight junction proteins

The quantitative real-time PCR technique was utilized to determine the mRNA expression levels of interleukin-1beta (*IL-1β*), *IL-6*, *IL-10*, tumor necrosis factor-alpha (*TNF-α*), *claudin-1*, *claudin-4*, *occludin* and zonula occludens-1 (*ZO-1*) in the small intestinal mucosa. Firstly, the total RNA was isolated from the duodenal, jejunal and ileal tissues using Trizol Reagent (Thermo Fisher Scientific, Inc., Waltham, United States). After detection of concentration and purity, the cDNA was reversely transcribed from isolated RNA by a reverse transcription kit. The expression levels of selected genes were measured using real-time PCR that was performed using the CFX PCR detection system (Bio-Rad Inc., Hercules, CA, United States) with fluorescence detection of SYBR green kit (Thermo Fisher Scientific, Inc., Waltham, United States). The real-time PCR was conducted in a reaction system as presented in Supplementary Table S2. Amplification primers of candidate genes were designed by utilizing primer 5.0 software, and primers were synthesized by Sangon Biotech Co., Ltd. (Shanghai, China). The information of primes are listed in Supplementary Table S3. In this study, the results of candidate gene expressions were analyzed by the 2^− ΔΔCt^ method (23) using the *β-actin* as housekeeping gene.

### 2.6 Statistical analysis

After checking of normality and homogeneity of variances tests, the data were analyzed by the one-way ANOVE procedures with each animal serving as an experimental unit in the SPSS software (version 22.0). The Duncan test was utilized to evaluate the differences among three groups. Results were presented as means and standard error of means. *p* < 0.05 was regarded as significant difference, and tendency for different groups was defined as 0.05 < *p* < 0.10.

## 3 Results

### 3.1 Serum permeability parameter

**Figure 1:**
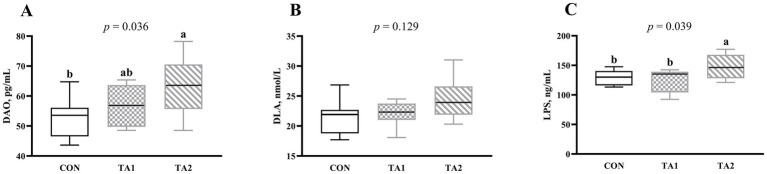
Effects of tannin supplementation on serum permeability parameters of fattening lambs. (A) DAO, diamine oxidase; (B) D-LA, D-lactic acid; (C) LPS, lipopolysaccharide. CON, TA1 and TA2 were fattening lambs fed basal diet and supplemented with tannin at the dosage of 0, 3 and 6 g/d per animal, respectively. Columns with different small letters differ significantly (*p* < 0.05).

As illustrated in Figure 1A, the DAO concentration in TA2 group was higher (*p* < 0.05) than that in CON group. There was no obvious difference (*p* > 0.05) of serum DLA content among all groups (Figure 1B). Compared with CON and TA1 groups, the LPS concentration in serum of TA2 group was increased (*p* < 0.05) by 14.44 and 19.12%, respectively (Figure 1C).

### 3.2 Slaughter performance

Obviously, the liveweight of lambs in TA1 group was higher (*p* < 0.05) than that in CON and TA2 groups (Table 1). Likewise, compared with CON group, the carcass weight of TA1 group was significantly increased (*p* < 0.05). No significant difference (*p* > 0.05) of meat percentage was found among all groups. However, the dressing percentage (*p* = 0.068) and meat weight (*p* = 0.077) of TA1 group tended to be increased by 5.40 and 7.38%, respectively, when compared to those of CON group.

**Table 1:** Effects of tannin supplementation on slaughter performance of fattening lambs.

Items	Treatments			SEM	*p*- value
	CON	TA1	TA2		
Liveweight (kg)	33.96^b^	35.11^a^	34.29^b^	0.157	0.004
Carcass weight (kg)	15.41^b^	16.80^a^	16.03^ab^	0.193	0.007
Dressing percentage (%)	45.38	47.83	46.74	0.440	0.068
Meat weight (kg)	12.06	12.95	12.26	0.170	0.077
Meat percentage (%)	35.53	36.86	35.78	0.444	0.449

SEM, standard error of mean. CON, TA1 and TA2 were fattening lambs fed basal diet and supplemented with tannin at the dosage of 0, 3 and 6 g/d per animal, respectively. Means with different superscript letters in the same row differ significantly (*p* < 0.05).

### 3.3 Meat quality

As shown in Table 2, the meat quality parameters including pH_45min_, pH_24h_, a*, b*, drip loss and cooking loss were similar (*p* > 0.05) among three treatments. Dietary supplementation of tannin significantly increased (*p* < 0.05) the IMF content of lambs. Nevertheless, an opposite trend of shear force was observed between the CON and tannin groups. In addition, the L* value of CON group was slightly higher (*p* = 0.060) than that of TA2 group.

**Table 2:** Effects of tannin supplementation on meat quality of fattening lambs.

Items	Treatments			SEM	*p*-value
	CON	TA1	TA2		
pH_45min_	6.65	6.82	6.73	0.035	0.176
pH_24h_	5.66	5.62	5.49	0.034	0.128
L*	37.25	35.51	35.29	0.376	0.060
a*	13.27	14.68	14.72	0.336	0.134
b*	11.68	12.02	12.21	0.183	0.498
IMF (%)	1.27^b^	1.73^a^	1.63^a^	0.061	0.002
Drip loss (%)	9.14	9.22	8.76	0.221	0.679
Cooking loss (%)	28.95	27.36	27.63	0.404	0.236
Shear force (N)	50.53^a^	45.31^b^	44.94^b^	1.046	0.043

L*, lightness, a*, redness, b*, yellowness, IMF, intramuscular fat, SEM, standard error of mean. CON, TA1 and TA2 were fattening lambs fed basal diet and supplemented with tannin at the dosage of 0, 3 and 6 g/d per animal, respectively. Means with different superscript letters in the same row differ significantly (*p* < 0.05).

### 3.4 Digestive enzyme activity

In the duodenum and ileum, dietary supplementation of tannin did not affect (*p* > 0.05) the activities of *α*-amylase, chymotrypsin and lipase (Table 3). Likewise, the lipase activity in the jejunum was similar (*p* > 0.05) among all groups. However, the trypsin activity in the jejunum and ileum of TA1 group was higher (*p* < 0.05) that that of CON and TA2 groups. Compared with CON group, the activity of jejunal α-amylase in TA1 group was increased (*p* < 0.05) by 14.78%. Additionally, the activities of duodenal trypsin (*p* = 0.073) as well as jejunal chymotrypsin (*p* = 0.059) in TA1 group were slightly higher than those in CON and TA2 groups, respectively.

**Table 3:** Effects of tannin supplementation on small intestinal digestive enzyme activity of fattening lambs (U/g).

Items	Treatments			SEM	*p*-value
	CON	TA1	TA2		
Duodenum					
α-amylase	15.97	14.81	16.79	0.941	0.708
Trypsin	143.38	158.68	156.23	2.981	0.073
Chymotrypsin	239.17	268.36	245.89	8.417	0.348
Lipase	42.76	40.52	39.45	2.051	0.812
Jejunum					
α-amylase	34.04^b^	39.07^a^	36.19^ab^	0.784	0.024
Trypsin	238.21^b^	264.70^a^	237.03^b^	5.196	0.041
Chymotrypsin	304.90	324.77	290.17	6.082	0.059
Lipase	110.74	101.14	95.76	4.819	0.457
Ileum					
α-amylase	26.41	30.17	31.34	0.995	0.103
Trypsin	249.14^b^	299.94^a^	264.87^b^	7.701	0.015
Chymotrypsin	392.49	421.26	389.61	9.086	0.303
Lipase	146.32	132.21	138.16	2.911	0.138

SEM, standard error of mean. CON, TA1 and TA2 were fattening lambs fed basal diet and supplemented with tannin at the dosage of 0, 3 and 6 g/d per animal, respectively. Means with different superscript letters in the same row differ significantly (*p* < 0.05).

### 3.5 Antioxidative status of small intestine

In the duodenum, lambs fed tannin had significantly elevated (*p* < 0.05) GSH-Px content (Table 4). The MDA concentration of TA1 group was lower (*p* < 0.05) than that of CON group. Moreover, the T-AOC level of TA1 group showed an increased trend (*p* = 0.071) when compared to that of CON group. A similar trend of GSH-Px level was observed between the CON and TA1 groups in the jejunum. Dietary supplementation of tannin significantly enhanced (*p* < 0.05) the SOD and T-AOC concentrations, whereas an opposite tendency of MDA was found between the CON and tannin supplementation groups. In the ileum, no obvious difference (*p* > 0.05) of GSH-Px and SOD levels was found among all groups. However, the addition of tannin significantly increased (*p* < 0.05) the T-AOC concentration of lambs. Besides, compared with CON group, the MDA concentration in TA1 group was decreased (*p* < 0.05) by 15.44%.

**Table 4:** Effects of tannin supplementation on antioxidative enzyme concentrations in the small intestine of fattening lambs.

Items	Treatments			SEM	*p*-value
	CON	TA1	TA2		
Duodenum					
GSH-Px (U/g)	61.95^b^	70.74^a^	72.66^a^	1.782	0.024
SOD (U/g)	124.72	115.77	120.74	2.378	0.320
MDA (nmol/g)	14.41^a^	12.13^b^	12.93^ab^	0.343	0.015
T-AOC (mmol/g)	0.415	0.535	0.484	0.022	0.071
Jejunum					
GSH-Px (U/g)	80.04	91.59	88.57	2.059	0.052
SOD (U/g)	101.21^b^	109.14^a^	111.61^a^	1.625	0.016
MDA (nmol/g)	21.31^a^	17.53^b^	17.21^b^	0.698	0.020
T-AOC (mmol/g)	0.571^b^	0.853^a^	0.919^a^	0.054	0.014
Ileum					
GSH-Px (U/g)	102.17	107.47	108.20	2.612	0.609
SOD (U/g)	135.50	130.68	136.85	3.538	0.772
MDA (nmol/g)	28.57^a^	24.16^b^	25.93^ab^	0.744	0.044
T-AOC (mmol/g)	0.311^b^	0.493^a^	0.459^a^	0.026	0.004

GSH-Px, glutathione peroxidase; SOD, superoxide dismutase; MDA, malondialdehyde; T-AOC, total antioxidant capacity; SEM, standard error of mean. CON, TA1 and TA2 were fattening lambs fed basal diet and supplemented with tannin at the dosage of 0, 3 and 6 g/d per animal, respectively. Means with different superscript letters in the same row differ significantly (*p* < 0.05).

### 3.6 Inflammatory factor expression in the small intestine

**Figure 2:**
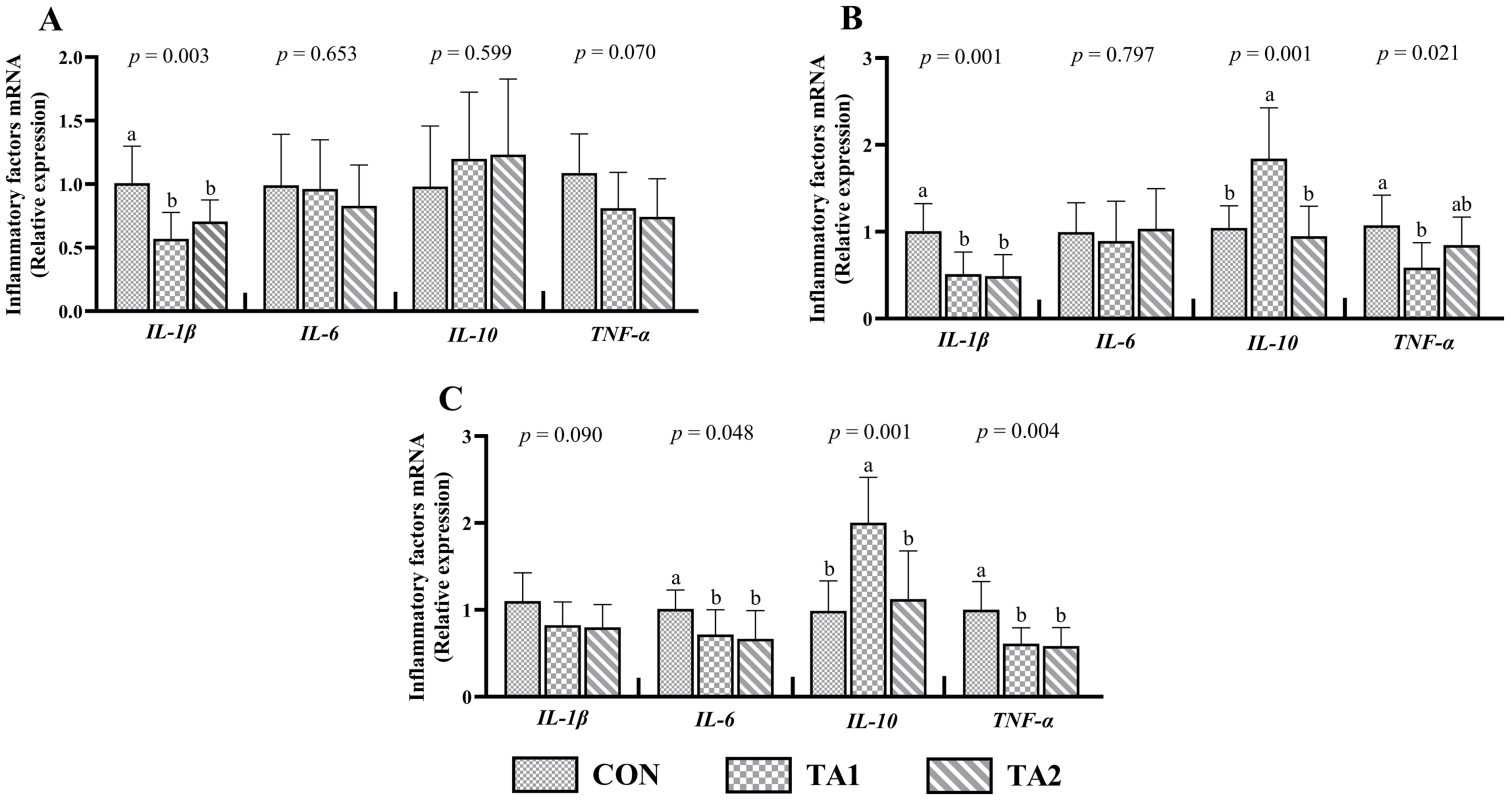
Effects of tannin supplementation on gene expression of inflammatory factors in the duodenum (A), jejunum (B) and ileum (C) of fattening lambs. *IL-1β*, interleukin-1beta; *IL-6*, interleukin-6; *IL-10*, interleukin-10; *TNF-α*, tumor necrosis factor-alpha. CON, TA1 and TA2 were fattening lambs fed basal diet and supplemented with tannin at the dosage of 0, 3 and 6 g/d per animal, respectively. Columns with different small letters differ significantly (*p* < 0.05).

Dietary supplementation with tannin significantly decreased (*p* < 0.05) the *IL-1β* mRNA levels in the duodenum (Figure 2A) and jejunum (Figure 2B). A similar trend of *IL-6* and *TNF-α* expressions between the CON and tannin groups was found in the ileum (Figure 2C). Also, the gene expression of jejunal *TNF-α* in TA1 group was lower (*p* < 0.05) than that in CON group. In the jejunum and ileum, the TA1 group had obviously up-regulated (*p* < 0.05) *IL-10* expression level as compared with CON and TA2 groups. The gene expressions of *IL-6* and *IL-10* in the duodenum as well as *IL-6* expression in the jejunum did not show significant difference (*p* > 0.05) among three treatments. In addition, lambs in TA2 group tended to have lower gene expressions of *TNF-α* (*p* = 0.070) in the duodenum and *IL-1β* (*p* = 0.090) in the ileum when compared to CON group.

### 3.7 Secretory immunoglobulin a concentration

**Figure 3:**
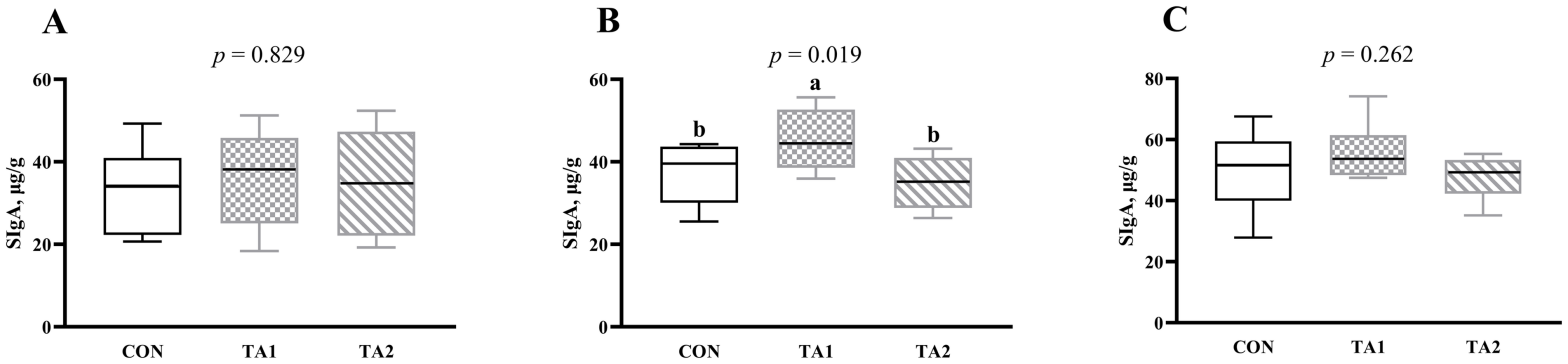
Effects of tannin supplementation on SIgA concentration in the duodenum (A), jejunum (B) and ileum (C) of fattening lambs. SIgA, secretory immunoglobulin A. CON, TA1 and TA2 were fattening lambs fed basal diet and supplemented with tannin at the dosage of 0, 3 and 6 g/d per animal, respectively. Columns with different small letters differ significantly (*p* < 0.05).

Notably, the SIgA concentration in the duodenum (Figure 3A) and ileum (Figure 3C) did not change (*p* > 0.05) with supplementation of tannin. However, compared with CON and TA2 groups, the SIgA concentration in the jejunum of TA1 group was increased (*p* < 0.05) by 20.48 and 29.89%, respectively (Figure 3B).

### 3.8 Tight junction protein expression in the small intestine

**Figure 4:**
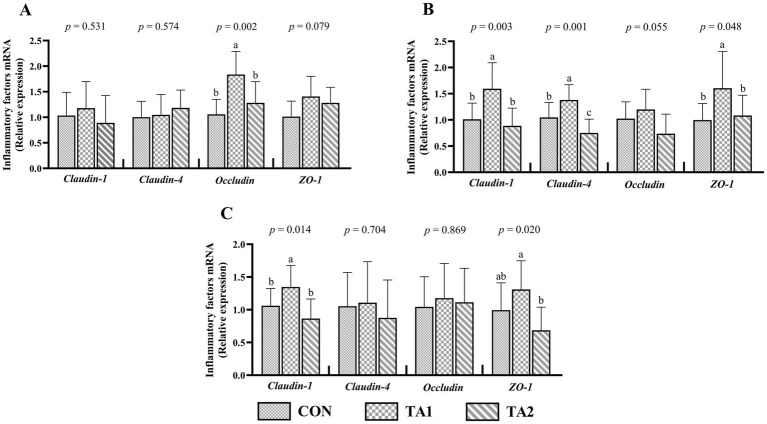
Effects of tannin supplementation on gene expression of tight junction proteins in the duodenum (A), jejunum (B) and ileum (C) of fattening lambs. *ZO-1*, zonula occludens-1. CON, TA1 and TA2 were fattening lambs fed basal diet and supplemented with tannin at the dosage of 0, 3 and 6 g/d per animal, respectively. Columns with different small letters differ significantly (*p* < 0.05).

In the duodenum, no obvious difference (*p* > 0.05) of *claudin-1* and *claudin-4* mRNA levels was found among three groups (Figure 4A). A similar pattern of *claudin-4* and *occludin* expressions was observed in the ileum (Figure 4C). Nevertheless, the *occludin* expression in the duodenum of TA1 group was higher (*p* < 0.05) than that of CON and TA2 groups. Compared with CON group, the duodenal *ZO-1* expression was increased (*p* = 0.079) by 39.60% in TA1 group. In the jejunum, the lambs in TA1 group had significantly increased (*p* < 0.05) *claudin-1*, *claudin-4* and *ZO-1* expression levels when compared to those in other two groups (Figure 4B). Furthermore, the mRNA abundance of *occludin* in TA1 group was slightly higher (*p* = 0.055) than that in TA2 group. In the ileum, compared with CON and TA2 groups, the *claudin-1* expression level was significantly up-regulated (*p* < 0.05) in TA1 group. In addition, the *ZO-1* expression of TA2 group was markedly down-regulated (*p* < 0.05) when compared to that of TA1 group.

## 4 Discussion

In the present sheep production, more and more attention has been paid to the lambs nutrition. The healthy feeding of fattening lambs has significant long-term influence on meat quality and productivity of sheep industry (24). In recent years, one of the important characteristics in the mutton market is its elevated requirement in term of meat quality, which promotes the rapid development of fattening lambs. Under the pattern of intensive house feeding, high-concentrate ration is commonly provided to sheep to maintain their growth and production performance. However, the high-concentrate diet induces several disorders including gastrointestinal dysfunction, which leads to an accumulation of endotoxins in the gastrointestinal tracts and the occurrence of oxidative stress as well as local inflammation, thereby reducing the production performance of animals (25). As a natural antioxidant, tannin possesses a variety of physiological functions, such as scavenging activity of free radical, inhibition of lipid peroxidation and alleviation of inflammatory response (8). An increasing number of studies have shown that hydrolysable tannin from plants can replace antibiotics and growth-promoting additives in animal feed (10, 26). In the current experiment, the liveweight and carcass weight of fattening lambs were significantly increased after dietary addition of tannin at the dosage of 3 g/d per animal. A recent research in ram lambs found that dietary supplementation with *Acacia mearnsii* tannin had no significant effect on liveweight during the experimental period (27). The result differences between our study and previous experiment may be that concentrate ratio in the diet of animals was distinct. Hydrolysable tannin can inhibit the process of bacterial biohydrogenation, increase the accumulation of beneficial fatty acids such as unsaturated fatty acids and reduce the production of saturated fatty acids by regulating the ruminal microbial community (28), which have positive effects on production performance of animals.

Slaughter performance can be used to evaluate the economic benefit of animals. Dressing and meat percentage can directly reflect the meat production performance of animals. Carcass and meat weights are important indexes used to assess the level of meat production (29). Compared with CON group, the carcass and meat weights were improved in TA1 group, indicating that lambs fed tannin can promote the deposition efficiency of nutrients. A previous study in finishing lambs reported that plant polyphenols had the ability to improve the meat production (30), which was basically in accordance with our study. In assessment of meat quality, higher pH means an increased water retention capacity (31). The changes of meat pH could be attributed to variation of glycogen concentration in muscles. With the reduction of meat pH, the polypeptide chain of protesin molecules become tight and the molecular spacing narrows, which squeezes the water molecules in the muscle, leading to reduced meat quality (22). In this experiment, no obvious difference of meat pH was observed among all groups, which was consistent with previous study in crossbred lambs for the addition of *Acacia mearnsii* tannin (32). However, the pH_24h_ value of TA2 group was numerically lower than that of CON group, suggesting that the suitable supplementation level should be less than 6 g/d per lamb.

Previously, a study in lambs found that there were an elevation of L* and reductions of a* and b* after dietary supplementation of tannin (33). The protective ability of tannin against myoglobin oxidation decreases the formation of posterior myoglobin and increases the concentration of heme, which can affect the regulation of meat color. Unfortunately, we did not find significant difference in meat color parameters after tannin supplementation. The reason for this difference may be that the materials used in the study were different (condensed tannins VS. hydrolysable tannin). However, our result showed that dietary supplementation of tannin significantly increased IMF content, which indicated that tannin supplementation could improve the tenderness of mutton under high-concentrate feeding model. The IMF content is an important index to affect the characteristics of meat such as juiciness and flavor because the accumulation of IMF can decrease collagen cross-linking to enhance tenderness of meat (30). This positive effect was further verified by shear force result that tannin supplementation significantly reduced the shear force of meat. Shear force is negatively correlated with meat tenderness. A recent study in lambs found that dietary supplementation with different proportions of chestnut tannin (0.3 and 0.6%) obviously reduced the meat shear force (34), which was in line with our finding. The possible reason may be that tannin can affect the arrangement mode of collagen fiber in the connective tissue and reduce the content of soluble collagen during the development of lambs. But the specific mechanism of action requires further investigated. Overall, high-concentrate supplemented with tannin can improve production performance of fattening lambs. In general, the improved production performance is closely related to intestinal function. Therefore, we conducted the following experiment to explore the influence of tannin on digestive enzyme activity, antioxidant ability and barrier function of small intestine in lambs.

The digestive enzyme in the gastrointestinal tracts determines the nutrient digestibility of animals. Condensed tannin can combine dietary starch, protein and digestive enzymes to form insoluble complexes, which reduce the palatability and nutrients digestibility of feed (8). Our study showed that supplementation of tannin at the dose of 3 g/d per lamb increased the activity of *α*-amylase, trypsin and chymotrypsin in the small intestine, indicating that appropriate amount of tannin supplementation promoted the digestion and absorption of nutrients in lambs. However, higher addition level reduced the trypsin activity of the small intestine, which had negative influence on crude protein digestibility (TA1 vs. TA2: 68.27% vs. 62.92%, *p* = 0.028; unpublished data). The reason could be partly attributed to that overmuch tannin combine with digestive enzymes in the gut to form inactive complexes, thus affecting the digestion and absorption of nutrients (8). But the potential mechanism of action should be further studied using *in vivo* and *in vitro* experiments. After long-term feeding of high-concentrate, the pH of intestine often will be decreased, and subsequently the activities of digestive enzymes are reduced, which lead to reproduction of harmful microorganisms and impair the intestinal function of animals (35). Our results verified that lambs receiving appropriate tannin can reverse the negative effect on digestive enzyme caused by high-concentrate feeding.

Lambs fed high-concentrate diet will be exposed to oxidative stress, which influences the cellular homeostasis and causes cell apoptosis, resulting in an adverse impact on the gut function (36). MDA is deemed to be as the metabolites of lipid peroxidation, and is the important index for oxidative stress. In this study, the MDA concentration of TA1 group in the duodenum, jejunum and ileum was lower than that of CON group, indicating that tannin can alleviate the small intestinal oxidative injury of lambs induced by high-concentrate feeding. A previous study in finishing lambs has found that lipid peroxidation had a negative influence on meat quality (30). Dietary supplementation of tannin enhanced the small intestinal SOD, GSH-Px and T-AOC activities to a certain degree, which suggested that tannin supplementation was conducive to improving the meat quality of lambs. A previous study in pre-puberty lambs had reported that the addition of tannin can enhance the antioxidant protection, inhibit the process of oxidation–reduction and protect the testis from damage by increasing the concentrations of antioxidant enzymes including T-AOC and SOD (37), which was basically consistent with our finding. When the tissue is subjected to oxidative stress, hydrolysable tannin can block the ubiquitination of *Nrf2*, promote the binding of *Nrf2* translocation with antioxidant response element and up-regulate the expression of a series of protective genes, including GSH and SOD, and then inducing the expression of more antioxidant enzymes in cells and down-regulating the production of reactive oxygen, finally enhancing the antioxidant capacity (38). In the future, the intestinal epithelial cells can be used as the research objective to explore the effects of tannin on antioxidant ability of gut. Moreover, oxidative stress can trigger intestine-related diseases, such as induction of oxidative damage to protein and DNA and elevation of intestinal epithelial permeability (36). Therefore, the positive influence of tannin on intestinal antioxidant function may be beneficial for improving intestinal barrier function.

Although the production performance of animals can be improved, long-term high-concentrate feeding damages the integrity of tight junction of the gut epithelium (35). The damaged epithelium results in increased intestinal permeability, which enables the hazardous substances, including pathogens and endotoxin, to enter into peripheral blood from intestinal lumen, thus causing local and systemic inflammation (39). Therefore, improving the gut barrier function will be conducive to healthy growth of lambs since it plays an essential role in maintaining physiological function of body. We firstly analyzed the changes of permeability parameters in serum of lambs after tannin supplementation. The DAO, existed in the epithelial villus of intestine, is commonly utilized to evaluate the permeability of intestinal epithelium, and increased DAO concentration of serum reflects impaired barrier function of intestine (5). Our experiments showed that lambs supplemented with higher level of tannin obviously increased the DAO activity in serum, indicating that overmuch tannin has adverse effect on intestinal epithelial permeability. When LPS translocates from the intestinal lumen into blood circulation, it damages the intestinal barrier and causes inflammatory response (5). In this study, higher level of tannin supplementation increased the serum LPS concentration. At present, the information of effects of tannin on serum permeability parameters in ruminants is limited. According to the serum results from current study, the supplementation level of tannin should be less than 6 g/d per lamb.

Even though the small intestine is critical to lambs since it is an important organ for digesting and absorbing nutrients as well as maintaining immunity, the small intestine receives much less attention relative to feed fermented organs (rumen and large intestine) in high-concentrate feeding studies of ruminants (4, 25). In young ruminants, previous study found that the improvement of gut tight junction was beneficial for reducing the incidence rate of some diseases such as diarrhea (40). Tannin can bind and coagulate the intestinal mucosal proteins, cause the intestinal mucosal surface to shrink and reduce the inflammatory content exudation and intestinal peristalsis frequency, which can enhance the intestinal function (41). In our study, tannin supplementation showed different effects on tight junction protein expressions in the duodenum, jejunum and ileum. Dietary supplementation of tannin at 3 g/d per lamb significantly increased the *occludin* expression in the duodenum, the *claudin-1* and *ZO-1* expressions in the jejunum and *claudin-1* and *ZO-1* expressions in the ileum, suggesting that appropriate addition of tannin can enhance the intestinal barrier function of lambs under high-concentrate feeding. Recently, Wang et al. (42) found that tannic acid supplementation could up-regulate the *ZO-1* expression in the jejunum, but it did not have significant effects on *occludin* and *claudin-1* expressions in the jejunum and ileum. The reason of difference in research findings between previous experiment and our research may be that the different intestinal segment respond differently to tannin. In addition, the positive influence of tannin on intestinal antioxidant ability was also conducive to improving barrier function of small intestine.

The SIgA in the intestine is an immunoglobulin secreted by mature plasma cells in the intestinal mucosa, and the SIgA released into the intestinal cavity can inhibit the adhesion of intestinal pathogens to the surface of the intestinal mucosa, thereby preventing them from damaging intestinal health (43). In the current study, the SIgA concentration in the jejunum of TA1 group was higher than that of CON and TA2 groups, which indicated that tannin was helpful for maintaining intestinal homeostasis. A previous study in piglets found that dietary supplementation of 0.1% tannic acid increased the intestinal SIgA content (44), which was consistent with our experiment. The pathways of T cell-dependent and T cell-independent that are influenced by intestinal microbial community are the main ways to regulate SIgA production (43). An *in vitro* experiment has found that tannin product had the ability to affect the composition of digestive microbial community (45). In the follow-up study, the effects of tannin on intestinal microbiota of lambs should pay more attention.

Intestinal inflammation is commonly accompanied by disruption of epithelial barrier function. Long-term high-concentrate feeding impairs the intestinal tight junction and causes the tissue inflammation (4). Our results displayed that the mRNA expression levels of pro-inflammatory factors *IL-1β*, *IL-6* and *TNF-α* were down-regulated and the anti-inflammatory factor *IL-10* was up-regulated in the small intestine of fattening lambs fed high-concentrate after tannin supplementation, suggesting the improvement of intestinal inflammation induced by tannin, which was conducive to improving the production performance as seen in the results of slaughter performance and meat quality. Previously, a study in broiler chickens found that dietary supplementation of tannin had no obvious effect on intestinal *IL-1β* and *IL-6* mRNA expressions, but increased the *ZO-1* expression (46). Tannin products have been found to block the activation of *MAPK* signaling pathway and alleviate inflammation by down-regulating the phosphorylation levels of four *MAPK* subfamilies, namely *JNK*, *ERK*, *p38* and *ERK5* (47). In addition, a recent study used intestinal epithelial cells as research objective and verified that tannin supplementation can attenuate the intestinal inflammation induced by *Enterotoxigenic Escherichia coli*, which was reflected in the reduction of pro-inflammatory cytokines *TNF-α* and *IL-1β* expressions by inhibiting the *TLR-4*-mediated *NF-κB* signaling pathway (48). We speculated that the positive influence of tannin on the improvement of intestinal inflammation in fattening lambs fed a high-concentrate diet may be related to suppression of *MAPK* and *NF-κB* signaling pathways. Nevertheless, in ruminants, the molecular mechanism of tannin on gut inflammation still requires further exploration. To sum up, the findings of this study showed that tannin was beneficial to enhance production performance of fattening lambs fed a high-concentrate diet by improving intestinal function.

## 5 Conclusion

Results from the current experiment provide evidence that of the addition of tannin at the level of 3 g/d per animal increases the carcass weight and IMF content of meat, and decreases meat shear force of fattening lambs fed a high-concentrate diet. Moreover, fattening lambs receiving tannin supplementation contributes in various ways to their small intestinal function, including elevation of digestive enzyme activity, enhancement of antioxidant capacity, inhibition of inflammatory responses, promotion of SIgA secretion and improvement of barrier function. Overall, based on the results mentioned earlier, hydrolysable tannin can be utilized as an effective feed additive to improve the intestinal health in fattening lambs fed a high-concentrate diet.

## Data Availability

The raw data supporting the conclusions of this article will be made available by the authors, without undue reservation.
