# Supplementary material for: High-concentrate diet supplemented with hydrolysable tannin improves the slaughter performance, intestinal antioxidant ability and barrier function of fattening lambs
Source: Front Vet Sci. 2024 Oct 28;11:1464314. doi: 10.3389/fvets.2024.1464314 (PMC11551998; doi:10.3389/fvets.2024.1464314)
Supplement: Supplementary file 1 [file Table_1.docx]

Supplementary Material

**Table S1** Compositions and nutrient levels of experimental diet (DM basis).

| Ingredients, % | Content | Nutrient levels | Content |
| --- | --- | --- | --- |
| Corn | 40.87 | DM (%) | 91.12 |
| Soybean meal | 8.37 | CP (%) | 16.20 |
| Cottonseed meal | 7.45 | EE (%) | 3.26 |
| Wheat bran | 10.64 | NDF (%) | 29.32 |
| Limestone | 0.31 | ADF (%) | 15.60 |
| NaCl | 0.50 | Ca (%) | 0.84 |
| NaHCO_3_ | 0.96 | P (%) | 0.49 |
| CaHPO_4_ | 0.40 | ME^2^ (MJ/kg) | 9.94 |
| Premix^1^ | 0.50 |  |  |
| Alfalfa hay | 22.36 |  |  |
| Corn straw | 7.64 |  |  |

DM, dry matter; CP, crude protein; EE, ether extract; NDF, neutral detergent fiber; ADF, acid detergent fiber; ME, metabolizable energy.

^1^ The premix provided following per kilogram of diet: Fe 100 mg, Zn 60 mg, Mn 40 mg, Cu 10 mg, I 0.60 mg, Se 0.40 mg, Co 0.20 mg, VA 5000 IU, VD 1200 IU, VE 40 IU.

^2^ ME was a calculated value; the other nutritional levels were analyzed according to AOAC procedures.

**Table S2** The reaction system of real-time PCR.

| Reagents | Volume, μL |
| --- | --- |
| 2×Real PCR SYBR Green | 10.0 |
| Template cDNA | 1.0 |
| Forward primer (10 μM) | 0.4 |
| Reverse primer (10 μM) | 0.4 |
| ddH_2_O | 8.2 |
| Total | 20.0 |

**Table S3** Primers used for quantitative real-time PCR of lambs in the current experiment.

| Genes | Primer sequence (5’-3’) | GenBank ID | Amplicon length, bp |
| --- | --- | --- | --- |
| β-actin | F: TCCGTGACATCAAGGAGAAGC | NM_001009784.2 | 266 |
|  | R: CCGTGTTGGCGTAGAGGT |  |  |
| IL-1β | F: CGTCTTCCTGGGACGTTTTAG | NM_001009465.2 | 112 |
|  | R: CTGCGTATGGCTTCTTTAGGG |  |  |
| IL-6 | F: AGGAAAAAGATGGATGCTTCCA | NM_001009392.1 | 156 |
|  | R: GACCAGCAGTGGTTTTGATCAA |  |  |
| IL-10 | F: CTCTGTGACGTATTTGGGAAGAA | [NM_001009327.1](https://www.ncbi.nlm.nih.gov/nuccore/NM_001009327.1) | 200 |
|  | R: GGTGAATGTGCCCTGTGAGTG |  |  |
| TNF-α | F: ACACCATGAGCACCAAAAGC | NM_001024860.1 | 168 |
|  | R: AGGCACAAGCAACTTCTGGA |  |  |
| Claudin-1 | F: AATACATTGAGGTCACCGAGTA | NM_001185016.1 | 191 |
|  | R: GATTAGGCAAGGAAAGGCAC |  |  |
| Claudin-4 | F: GCCTTCATCGGCAGCAACAT | NM_001185017.1 | 115 |
|  | R: CCAGCAGCGAGTCGTACACCTT |  |  |
| Occludin | F: AGTGGTAACTTGGAGACGCTTTC | XM_012145891.2 | 107 |
|  | R: CCTCCCGTCGTGTAGTCTGTT |  |  |
| ZO-1 | F: CATCACGCCAGCATACAA | XM_042235171.1 | 177 |
|  | R: GCAGACTTCAGGAGGGTTT |  |  |

IL-1β, interleukin-1beta; IL-6, interleukin-6; IL-10, interleukin-10; TNF-α, tumor necrosis factor-alpha; ZO-1, zonula occludens-1.

F, forward; R, reverse.
